# Association of viral load values with cervical lesion severity and exploratory genotype analysis in HPV-positive Indian women

**DOI:** 10.3389/fgwh.2026.1891484

**Published:** 2026-10-29

**Authors:** Nilanchali Singh, Zakiya Zakiya, Seema Singhal, Jyoti Meena, Rajesh Kumari, Anju Singh, Iman Dandapath, Anjli Gaur, Pranay Tanwar, Sandeep Mathur, Maroof Ahmad Khan, Neena Malhotra

**Affiliations:** 1 Department of Obstetrics and Gynaecology, All India Institute of Medical Sciences, New Delhi, India; 2 Department of Laboratory Oncology, Dr. B.R.A. Institute Rotary Cancer Hospital, All India Institute of Medical Sciences, New Delhi, India; 3 Department of Pathology, All India Institute of Medical Sciences, New Delhi, India; 4 Department of Biostatistics, All India Institute of Medical Sciences, New Delhi, India

**Keywords:** cervical cancer screening, cervical lesions, genotyping, human papillomavirus, hybrid capture 2, relative light unit/cutoff

## Abstract

**Background:**

Hybrid Capture 2 (HC2) provides a semi-quantitative estimate of high-risk human papillomavirus (HPV) burden as relative light units/cutoff (RLU/CO), and HPV genotyping may add value to triage assessment. We evaluated the association of RLU/CO with cervical lesion severity and explored whether genotype information improves diagnostic performance in HPV-positive Indian women.

**Methods:**

This cross-sectional study was conducted in two sequential phases. In the initial phase, 217 consecutive HPV-positive women underwent HC2 HPV DNA testing, colposcopy-guided biopsy, and histopathological assessment. In a subsequent phase, an additional group of 111 HPV-positive women underwent the same clinical and histopathological assessment and also provided specimens for HPV genotyping. The genotype-enriched group was analysed separately for genotype distribution, co-infection patterns, and exploratory genotype-integrated prediction models. Age, Histopathology, and viral load (HC2 RLU/CO) were analysed in the full cohort. The genotyped subset was additionally evaluated for HPV genotype distribution and genotype-integrated ROC models. Descriptive statistics, Kruskal–Wallis testing with Holm-adjusted Dunn's *post-hoc* comparisons, and ROC analyses with 95% confidence intervals and paired DeLong comparisons were performed.

**Results:**

RLU/CO values differed across study-specific histopathological severity categories, with higher median values in LSIL, HSIL, and invasive cervical cancer than in normal histology and chronic cervicitis. In the cohort of 328 women, RLU/CO discriminated HSIL or invasive cervical cancer (severity score ≥3) with an AUC of 0.798 (95% CI, 0.726–0.868). A study-derived Youden-index cutoff of 12.08 RLU/CO yielded a sensitivity of 81.1% (95% CI, 69.5%–90.9%) and specificity of 73.1% (95% CI, 68.0%–78.0%). Among 111 genotyped participants, HPV16 was the most frequently detected genotype, and multiple-genotype infections occurred in 64 participants (57.7%). In the genotyped subgroup, comparative ROC analysis for HSIL or invasive cervical cancer showed AUCs of 0.659 for RLU/CO alone, 0.608 for RLU/CO plus HPV16/18, and 0.461 for RLU/CO plus other high-risk HPV. Neither genotype-integrated model improved discrimination compared with RLU/CO alone; these exploratory estimates require validation in larger independent cohorts.

**Conclusion:**

HC2 RLU/CO showed potential for identifying HSIL or invasive cervical cancer. The study-derived cutoff requires validation in independent populations. Genotype-integrated models showed variable performance in the small subgroup. The HPV16/18 model was comparable to RLU/CO alone, but findings were exploratory. Larger prospective studies are needed before clinical implementation.

## 1 Introduction

Persistent high-risk human papillomavirus (HPV) infection is the central etiological factor for cervical intraepithelial neoplasia (CIN) and cervical cancer (1). However, not all HPV-positive women have the same risk of developing clinically significant cervical lesions, and triage tools are needed to distinguish women at low risk from those who require further evaluation. The Hybrid Capture 2 (HC2) assay is widely used for HPV screening, providing a relative light unit/cutoff (RLU/CO) value, which acts as a semi-quantitative measure of high-risk HPV viral load (1). Several studies indicate that higher RLU/CO values correlate with more severe cervical lesions, but standardised thresholds for clinical triage and predictive reliability remain undetermined (2–4). In addition, HPV genotype may influence the biological behaviour of infection, especially when HPV16/18 are present. Recent work suggests that combining viral load and genotyping can improve clinical triage over either marker alone (5).

Fobian et al. confirmed the positive relationship between viral load and lesion severity but noted significant heterogeneity among studies in cut-points and diagnostic utility (6). Zhou et al. reviewed the interplay between RLU/CO bands, genotype, and lesion outcome (2), while Chen et al. reported that quantitative stratification meaningfully improves cervical disease risk discrimination even in the absence of adjunct cytology/genotyping (3). However, most studies use arbitrary or poorly validated bands (e.g., <10, 10–50 RLU/CO), leaving a gap in evidence-based operational cutoffs for clinical use. While HPV-based screening improves case detection, a low cutoff for triage can increase colposcopy referrals and contribute to unnecessary workload and patient burden. Risk stratification strategies are therefore needed to identify women who should be prioritised for further evaluation (7).

There is a need for population-specific validation of RLU/CO stratification, using histopathology as the gold standard, and rigorous statistical analysis models. The present study was designed to evaluate the association and diagnostic performance of HC2 RLU/CO values for histopathological cervical lesion severity in HPV-positive women. The secondary objective was to describe HPV genotype distribution and explore whether genotype variables add discriminatory information to RLU/CO in a subsequently recruited genotype-enriched cohort.

## 2 Materials and methods

### 2.1 Study population and study design

This cross-sectional study enrolled 328 sexually active women of reproductive age who tested positive for high-risk human papillomavirus (HPV) infection by Hybrid Capture 2 assay (RLU/CO value >1). Participants were recruited at the Department of Obstetrics and Gynaecology, AIIMS New Delhi, between November 2022 and May 2025, following approval of the study protocol by the Institutional Ethics Committee and after obtaining informed consent (Ref. No.: IEC-828/04.11.2022). Recruitment occurred in two sequential phases: an initial phase of 217 HPV-positive women who underwent HC2 testing, colposcopy-guided biopsy, and histopathological assessment, followed by a subsequent phase of 111 HPV-positive women who underwent the same evaluation and additionally provided a separate cervical specimen for HPV genotyping.

Each patient received a patient information sheet, and written consent was obtained and documented for all participants. Following HPV testing, all enrolled women underwent colposcopy-guided cervical biopsy for histopathological evaluation as part of the institutional diagnostic pathway for HPV-positive women referred for evaluation, rather than as part of population-based primary screening. Women were eligible if they had a valid positive HC2 result and an available histopathological diagnosis. Participants without histopathological results or with inadequate biopsy specimens were excluded from the diagnostic-performance analysis. Genotyping was performed only in the second recruitment phase; samples with unsuccessful or missing genotyping results were excluded from genotype-specific analyses.

The full cohort was used to analyse RLU/CO and Study-specific histopathological severity score (Normal=0, Chronic Cervicitis = 1, LSIL = 2, HSIL = 3, Cancer = 4), based on histopathological findings. As HPV genotyping was introduced later in the study and thus was performed only in participants recruited during the genotype-testing phase for whom an additional cervical specimen was collected and suitable for molecular analysis. Consequently, genotype data were available for 111 participants. The genotype cohort was therefore not a randomly selected sample of the initial cohort and was analysed as an exploratory, temporally distinct cohort.

### 2.2 Specimen collection and molecular testing

#### 2.2.1 HC2 HPV DNA testing

Cervical samples for Hybrid Capture 2 (HC2) testing were collected using the HC2 Digene Qiagen cervical sampler (1 mL of Specimen Transport Medium STM). These cervical specimens were subsequently processed using HC2 reagents and kits from Qiagen, Hilden, Germany, to estimate high-risk HPV viral load semi-quantitatively as the relative light unit/cutoff (RLU/CO) ratio. The assay detects DNA from 13 high-risk HPV types: HPV16, 18, 31, 33, 35, 39, 45, 51, 52, 56, 58, 59, and 68. Each run included the manufacturer-provided positive and negative controls, and the run was accepted only when the control results met the specified validity criteria. Samples with RLU/CO ≥1.0 were considered positive according to the manufacturer's recommended threshold. Samples with invalid control results or technically inadequate results were repeated according to the laboratory's standard operating procedure. Very high RLU/CO values were recorded as reported by the assay and were not truncated or capped; where values exceeded the instrument's measurable range, the laboratory's validated reporting procedure was followed.

#### 2.2.2 HPV genotyping

For HPV genotyping, a separate cervical specimen was collected using a cervical sampling brush and transferred into a 5-mL BD SurePath liquid-based cytology (LBC) vial containing the manufacturer-provided preservative medium. Subsequently, HPV genotyping was performed by an external laboratory using a validated next-generation sequencing protocol. DNA extraction, library preparation, sequencing, genotype assignment, and quality-control procedures were conducted according to the laboratory's standard operating procedures. Genotypes were reported only when the laboratory-defined quality criteria were satisfied. Samples that did not meet the required quality criteria or for which sequencing was unsuccessful were recorded as missing genotype results and were excluded from genotype-specific analyses. Mixed infection was defined as detection of two or more HPV genotypes in the same specimen.

### 2.3 Histopathological assessment

All participants underwent colposcopy-guided cervical biopsy, and the specimens were evaluated by two experienced pathologists using standard histopathological criteria. The pathologists were blinded to the participants' HPV viral-load and genotype results at the time of histopathological assessment. Participants were classified according to a study-specific histopathological severity score derived from biopsy findings: score 0, normal histology or no evidence of dysplasia or malignancy; score 1, chronic cervicitis or benign inflammatory changes; score 2, low-grade squamous intraepithelial lesion (LSIL); score 3, high-grade squamous intraepithelial lesion (HSIL); and score 4, invasive cervical cancer. In cases of discordant interpretation, the specimens were reviewed jointly by the involved pathologists and a consensus diagnosis was reached; where consensus could not be achieved, the final classification was determined by a senior pathologist. Histopathology was used as the reference standard for all diagnostic analyses.

### 2.4 Data collection

The dataset included age, histopathology findings, and viral load values (HC2 RLU/CO). For the genotyped subset, HPV genotype information was obtained and grouped for analysis into HPV16/18, other high-risk HPV, and low-risk HPV categories. Mixed infections were also assessed.

### 2.5 Sample size calculation

Sample size was based on the availability of consecutive HPV-positive women undergoing colposcopy-guided biopsy during the study period, with the full cohort used for the primary diagnostic analysis of RLU/CO. The genotype subgroup was analysed exploratorily, as genotype data were available only in a subset of participants. The study was therefore sufficiently sized for the primary ROC analysis but not specifically powered for genotype-level subgroup comparisons.

### 2.6 Statistical analysis

Continuous variables were summarised as Mea*n* ± SD and median with IQR, while categorical variables were summarised as frequencies and percentages. Because RLU/CO values were strongly right-skewed, medians and IQRs were reported alongside means and SDs, and log10-transformed RLU/CO values were used for graphical presentation. Overall differences between histopathological categories were assessed using the Kruskal–Wallis test as RLU/CO distribution was non-normal. When the overall test was statistically significant, pairwise comparisons were performed using Dunn's *post-hoc* test. *P* values were adjusted for the 10 possible pairwise comparisons using the Holm method. Statistical significance was defined as a two-sided adjusted *P* < 0.05. Although age differed across histopathological categories, it was not included in the multivariable models because of the limited number of outcome events and the exploratory nature of the genotype-subgroup analysis.

ROC curves with 95% CI were constructed to evaluate the diagnostic performance of RLU/CO for predicting clinically significant lesions, defined as a Study-specific histopathological severity score ≥3. In the genotyped subset, AUCs were reported with 95% confidence intervals and compared using paired DeLong tests because all models were evaluated in the same participants. Exploratory logistic-regression models were fitted to predict HSIL or invasive cervical cancer (study-specific histopathological severity score ≥3). RLU/CO was entered as a continuous predictor and combined separately with HPV16/18 status or other high-risk HPV status. Each genotype indicator was coded as positive when the relevant genotype(s) were detected; indicators were not mutually exclusive in women with mixed infections. All analyses used SPSS v25.2; *p* < 0.05 was considered significant. Given the exploratory nature, absence of internal validation and small size of the genotyped subgroup, statistically significant AUC differences were interpreted cautiously and were not considered evidence of clinical validation.

## 3 Results

### 3.1 Baseline characteristics

The study cohort comprised 328 HPV-positive women with available HC2 RLU/CO and histopathological data. Baseline characteristics and RLU/CO distributions according to the study-specific histopathological severity categories are presented in Table 1. The histopathological severity-score distribution was as follows: normal, 117 (35.7%); chronic cervicitis, 140 (42.7%); LSIL, 18 (5.5%); HSIL, 20 (6.1%); and invasive cervical cancer, 33 (10.1%). The overall mean age was 41.16 ± 10.98 years, and the median age was 40 years (IQR, 32–49). The overall mean RLU/CO was 73.76 ± 228.48, with a median of 4.06 (IQR, 1.86–27.10) and a range of 1.00–1,666.42. RLU/CO values differed significantly across histopathological categories according to the Kruskal–Wallis test [H (4) = 54.35, *P* < 0.001]. Age also differed significantly across categories [H (4) = 15.77, *P* = 0.003].

**Table 1:** Characteristics and RLU/CO distribution by study-specific histopathological severity score (total *n* = 328).

Histopath. severity score	Histopathology findings	*N* (%)	Age Mean ± SD (Median, IQR)	RLU/CO Mean ± SD (Median, IQR)	RLU/CO Range (Min-Max)
0	Normal/no evidence of dysplasia or malignancy	117 (35.7%)	40.64 ± 11.82 (39.00, 31.00–50.00)	47.25 ± 205.79 (2.93, 1.75–10.07)	1.04–1,666.42
1	Chronic cervicitis or benign inflammatory changes	140 (42.7%)	40.10 ± 10.03 (39.00, 32.00–47.00)	42.21 ± 161.36 (2.88, 1.63–15.82)	1.00–1,100.20
2	LSIL/low-grade squamous intraepithelial lesion	18 (5.5%)	39.72 ± 11.75 (38.00, 32.25–45.75)	26.55 ± 32.05 (14.18, 3.07–31.98)	1.42–120.41
3	HSIL/high-grade squamous intraepithelial lesion	20 (6.1%)	49.15 ± 8.80 (50.00, 44.75–56.00)	242.04 ± 331.28 (123.15, 12.43–243.47)	2.61–1,043.00
4	Invasive cervical cancer	33 (10.1%)	43.39 ± 10.80 (44.00, 34.00–50.00)	225.41 ± 389.25 (32.35, 18.09–315.60)	1.06–1,467.77
Total		**328** (**100.0%)**	**41.16** **±** **10.98 (40.00, 32.00–49.00)**	**73.76** **±** **228.48 (4.06, 1.86–27.10)**	**1.00**–**1,666.42**

Baseline characteristics and HPV viral load (RLU/CO) distribution across histopathological categories in 328 HPV-positive Indian women who underwent colposcopy-guided cervical biopsy. Histopathological severity was classified using a study-specific score: 0, normal histology; 1, chronic cervicitis or benign inflammatory findings; 2, LSIL; 3, HSIL; and 4, invasive cervical cancer. RLU/CO values were highly right-skewed; therefore, medians and IQRs are reported in addition to means and standard deviations.

Bold values indicate the overall total-cohort values.

### 3.2 Viral load trend across study-specific histopathological severity score categories

RLU/CO values differed significantly across the five histopathological severity categories according to the Kruskal–Wallis test [H (4) = 54.35, *P* < 0.001]. Pairwise Dunn's *post-hoc* testing was performed for RLU/CO across the five histopathological severity groups. In the Dunn's *post-hoc* analysis with Holm adjustment (Table 2), RLU/CO values were significantly higher in the HSIL group than in the normal group (*P* < 0.001) and chronic cervicitis group (*P* < 0.001). Similarly, RLU/CO values were significantly higher in the cancer group than in the normal group (*P* < 0.001) and chronic cervicitis group (*P* < 0.001). Comparisons between normal and LSIL, chronic cervicitis and LSIL, LSIL and HSIL, LSIL and cancer, and HSIL and cancer were not statistically significant after adjustment for multiple comparisons (Table 2). Viral load (RLU/CO) values showed a wide right-skewed distribution; they were log10-transformed to improve visual interpretability and allow clearer comparison across histopathological severity score categories. The boxplot of log10(RLU/CO) demonstrated variation in viral load across the histopathological spectrum, with lower values in the normal and chronic cervicitis groups and higher values in the HSIL and cancer categories (Figure 1).

**Table 2:** Pairwise Dunn's *post-hoc* test for RLU/CO across the five histopathological severity groups.

Pairwise comparison	z-value	Unadjusted *P*-value	Holm-adjusted *P*-value
Normal vs. chronic cervicitis	0.176	0.861	0.861
Normal vs. LSIL	−2.389	0.017	0.084
Normal vs. HSIL	−4.969	<0.001	***<0***.001*
Normal vs. cancer	−4.938	<0.001	***<0***.001*
Chronic cervicitis vs. LSIL	−2.503	0.012	0.074
Chronic cervicitis vs. HSIL	−5.122	<0.001	***<0***.001*
Chronic cervicitis vs. cancer	−5.144	<0.001	***<0***.001*
LSIL vs. HSIL	−1.839	0.066	0.264
LSIL vs. cancer	−1.258	0.209	0.626
HSIL vs. cancer	0.808	0.419	0.838

Overall comparison was performed using the Kruskal–Wallis test. Pairwise comparisons were performed using Dunn's test, with Holm adjustment for 10 pairwise comparisons. Statistical significance was defined as an adjusted *P*-value <0.05, marked with an asterisk (*).

Bold values indicate statistically significant pairwise comparisons after Holm adjustment (adjusted *P* < 0.05).

**Figure 1:**
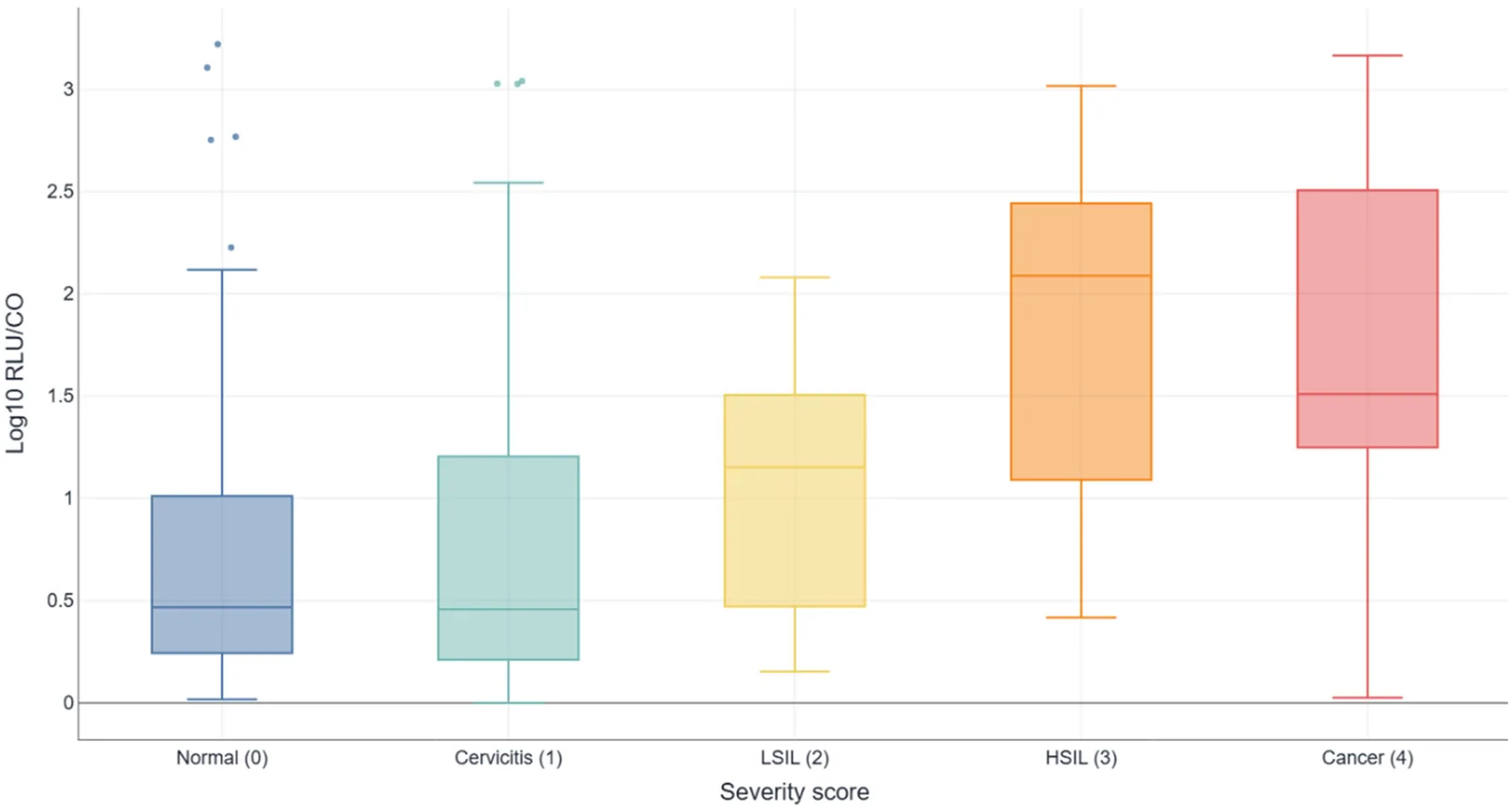
Boxplot showing distribution of log₁₀-transformed hybrid capture 2 RLU/CO values across histopathological categories. The analysis included 328 HPV-positive women: normal histology (*n* = 117), chronic cervicitis (*n* = 140), LSIL (*n* = 18), HSIL (*n* = 20), and invasive cervical cancer (*n* = 33). The boxes represent the median and interquartile range, whiskers extend to 1.5 times the IQR, and individual points represent outlying observations. RLU/CO distributions differed significantly across the five histopathological categories, with the greatest differences observed between benign categories and HSIL or cancer.

### 3.3 Diagnostic performance of viral load for clinically significant lesions

In the full cohort, viral load (RLU/CO) demonstrated good diagnostic performance for identifying clinically significant cervical lesions, defined as a Study-specific histopathological severity score ≥3 (HSIL and Cancer), with an AUC of 0.798 (95% CI, 0.726–0.868). The optimal Youden cutoff was 12.08 RLU/CO, which yielded a sensitivity of 81.1% *(95% CI, 69.5%–90.9%)* and a specificity of 73.1% *(95% CI, 68.0%–78.0%)* (Figure 2). These findings suggest that HC2 viral load can provide useful discrimination for triaging HPV-positive women at risk of significant cervical pathology.

**Figure 2:**
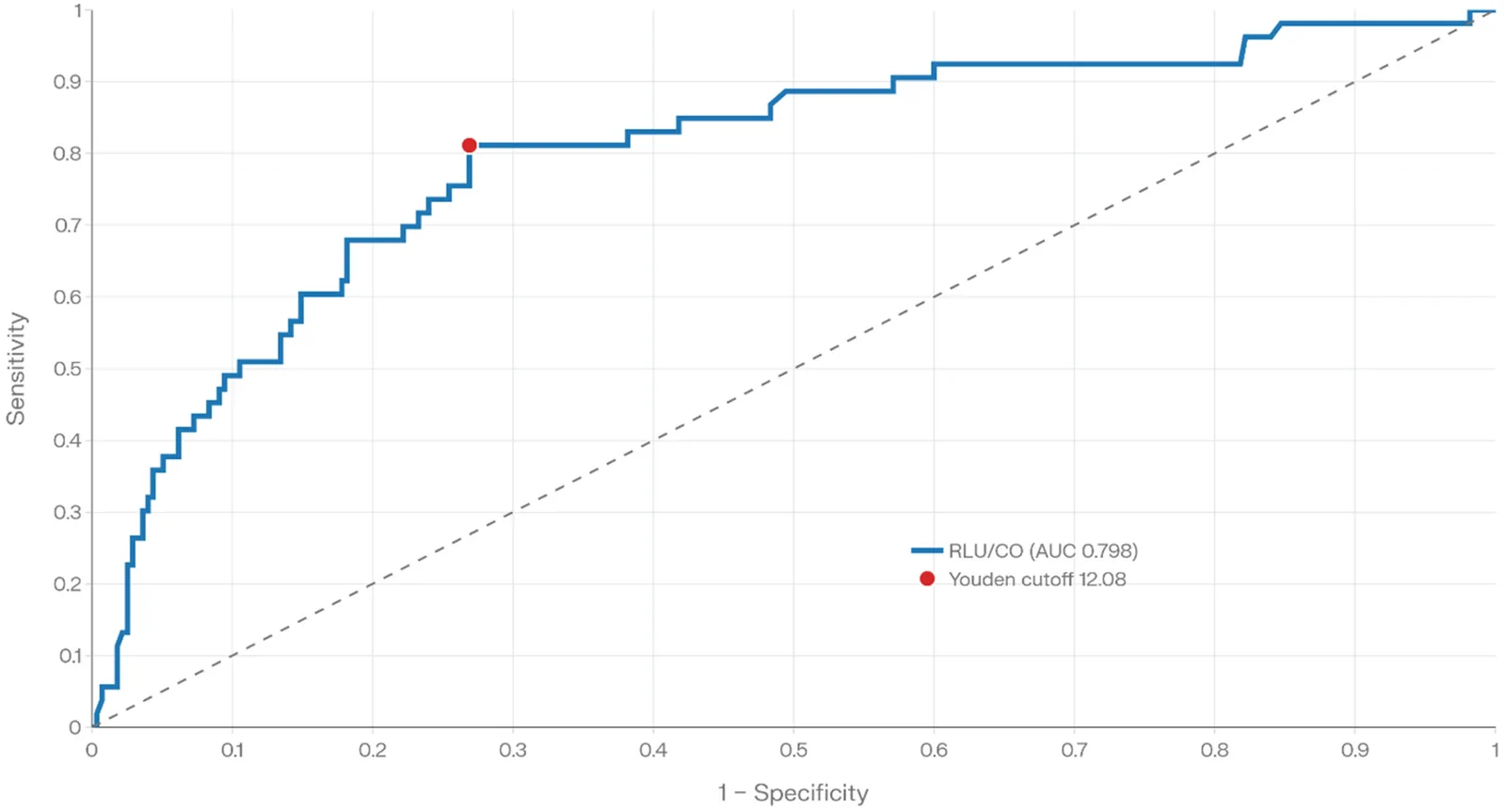
Receiver operating characteristic (ROC) curve for RLU/CO predicting HSIL and invasive cancer (study-specific histopathological severity score ≥3). Receiver Operating Characteristic (ROC) curve demonstrating the diagnostic performance of Hybrid Capture 2 RLU/CO values in predicting clinically significant cervical lesions (HSIL & Cancer; score of ≥3). The analysis included 328 HPV-positive women, including 53 participants with HSIL or invasive cervical cancer and 275 participants with a study-specific histopathological severity score <3. The blue curve represents RLU/CO discrimination, with an AUC of 0.798 (95% CI, 0.726–0.868). The red point indicates the study-derived Youden index cutoff of 12.08 RLU/CO, yielding sensitivity of 81.1% (95% CI, 69.5%–90.9%) and specificity of 73.1% (95% CI, 68.0%–78.0%). Because the cutoff was selected and evaluated in the same cohort, it should be considered exploratory.

### 3.4 HPV genotype spectrum in the genotyped subset (*n* = 111)

Among the 111 participants with available HPV genotyping results, 25 distinct HPV genotypes were detected. A total of 255 genotype detections were recorded, with 47 participants (42.3%) carrying a single genotype and 64 (57.7%) carrying multiple genotypes. HPV16 was the most frequently detected genotype, present in 58 participants (52.3%), followed by HPV31 in 39 (35.1%), HPV6 in 23 (20.7%), HPV42 in 16 (14.4%), HPV61 in 15 (13.5%), and HPV33 and HPV59 in 14 participants each (12.6%). HPV18 was detected in 8 participants (7.2%) (Figure 3a). Because multiple genotypes could be detected in the same participant, genotype-specific percentages were calculated using the 111 genotyped participants as the denominator.

**Figure 3:**
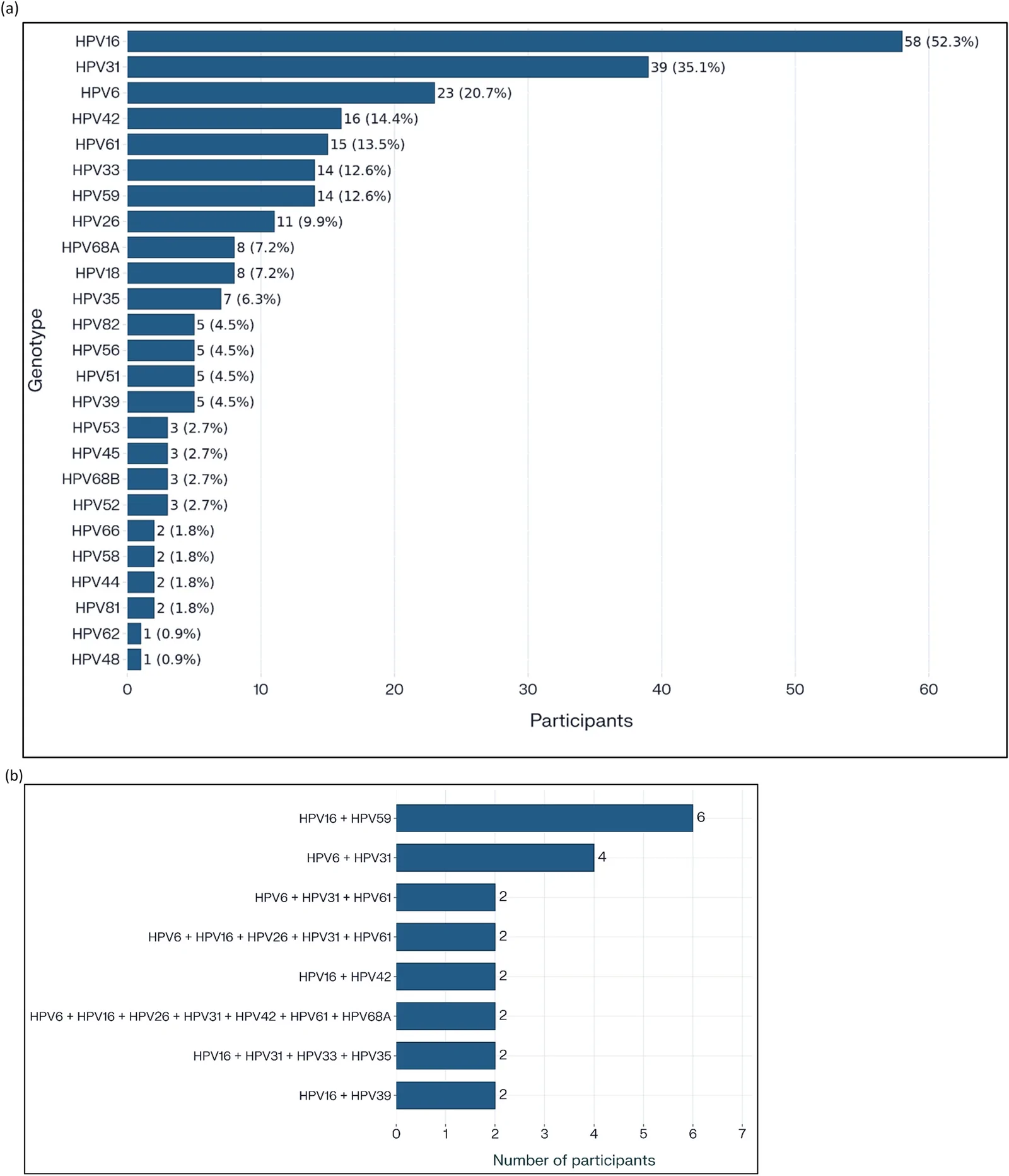

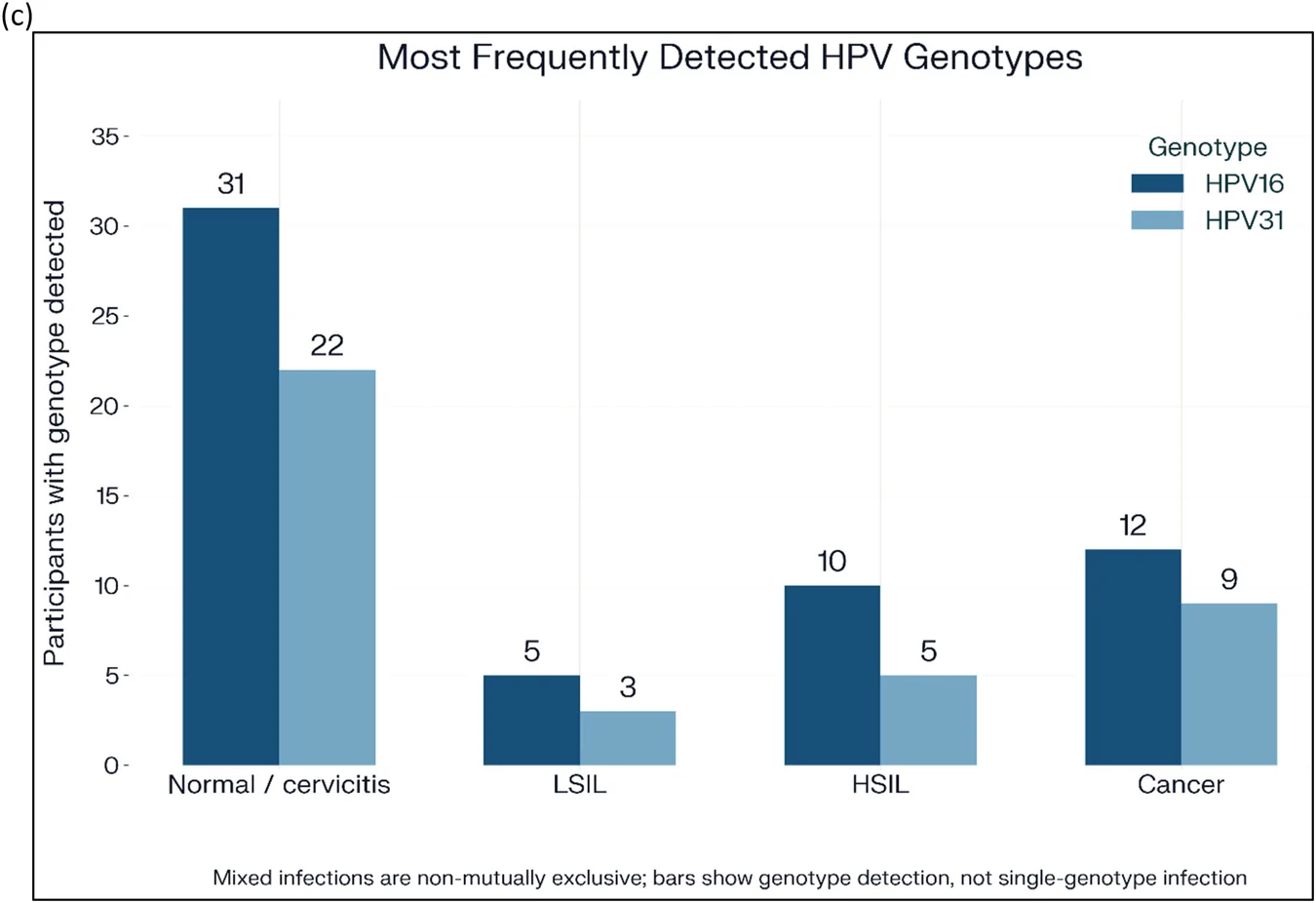
**(a)** Distribution and prevalence of detected HPV genotypes (*n* = 111), **(b)** Most frequent HPV co-infection combinations in the genotyped cohort, **(c)** The two most frequently detected HPV genotype across different histopathological categories (*n* = 111). **(a)** Bar graph illustrating the number of participants carrying each HPV genotype among 111 genotyped women; a total of 25 distinct HPV genotypes identified in the study population. Because multiple-genotype infections were common, participants could contribute to more than one genotype category. Percentages were calculated using 111 as the denominator. HPV16 and HPV31 emerge as the most frequently detected strains, accounting for a significant portion of the total infections. This spectrum highlights the high molecular diversity of HPV in this cohort, reinforcing the clinical importance of comprehensive genotyping for identifying high-risk infections beyond the common types. **(b)** Bar graph showing the most common HPV co-infection patterns observed among the 111 genotyped patients. The tallest bar represents HPV16 + HPV59 co-infection (*n* = 6), followed by HPV31 + HPV6 (*n* = 4). Several other genotype combinations occurred in smaller numbers, each affecting 2 patients. This figure highlights the diversity and frequency of mixed HPV infections in the cohort, underscoring the importance of comprehensive genotyping for accurate molecular characterisation and risk stratification. **(c)** Bars show the number of genotyped participants in whom HPV16 or HPV31 was detected within each histopathological category. The normal and chronic-cervicitis groups were combined as a benign reference category. Because participants could have multiple HPV genotypes, genotype detections were not mutually exclusive and do not represent single-genotype infections.

### 3.5 Co-infection patterns and genotype categories

Mixed HPV infection was common in the genotyped cohort, and several recurrent co-infection patterns were observed. Among the 64 participants with multiple-genotype infection, 50 distinct exact genotype patterns were identified. The most frequent pattern was HPV16 plus HPV59, detected in 6 participants, followed by HPV6 plus HPV31, detected in 4 participants (Figure 3b). Several other patterns occurred in two participants each, whereas most patterns were observed in only one participant. Because participants were classified according to their complete genotype profile, exact patterns were mutually exclusive and collectively accounted for all 64 participants with multiple-genotype infection.

### 3.6 Genotype distribution within study-specific histopathological severity score categories

HPV16 was the most frequently detected genotype in the HSIL and cancer groups, occurring in 10 of 11 HSIL cases and 12 of 17 cancer cases. HPV31 was also frequently detected in both groups (Figure 3b). HPV6 was detected in 5 HSIL cases and 6 cancer cases; however, genotype detections were generally evaluated in the context of mixed infections, and these counts do not represent HPV6-only infections.

Among the 111 genotyped participants, 30 (27.0%) had normal histology, 42 (37.8%) had chronic cervicitis, 11 (9.9%) had LSIL, 11 (9.9%) had HSIL, and 17 (15.3%) had invasive cervical cancer. Participants were assigned to mutually exclusive genotype categories according to the predefined classification hierarchy. Mixed-genotype infection was the most frequent category overall, occurring in 64 of 111 participants (57.7%). It was observed in 15/30 participants with normal histology (50.0%), 24/42 with chronic cervicitis (57.1%), 6/11 with LSIL (54.5%), 9/11 with HSIL (81.8%), and 10/17 with invasive cervical cancer (58.8%). The distribution of mutually exclusive genotype categories according to histopathological category is shown in Table 3.

**Table 3:** Mutually exclusive HPV genotype categories according to histopathological category.

Histopathology	HPV16-only	HPV18-only	Other high-risk-only	Low-risk only	Mixed Infections	No. of patients genotyped (*n* = 111)
Normal	4	0	7	4	15	30 (27.0%)
Chronic cervicitis	8	0	9	1	24	42 (37.8%)
LSIL	2	0	1	2	6	11 (9.9%)
HSIL	1	0	1	0	9	11 (9.9%)
Cancer	5	0	2	0	10	17 (15.3%)

Participants were assigned to one mutually exclusive genotype category using a predefined classification hierarchy. ‘HPV16-only’ and ‘HPV18-only’ indicate detection of the specified genotype without any additional genotype. ‘Other high-risk-only’ indicates detection of one high-risk genotype other than HPV16 or HPV18. ‘Low-risk/other-only’ indicates detection of one genotype not included in the high-risk group. ‘Mixed infection’ indicates detection of two or more genotypes, irrespective of genotype risk group.

### 3.7 Comparative ROC models in the genotyped cohort

Among the genotyped HPV-positive participants (*n* = 111), 28 had clinically significant histopathology, defined as a study-specific histopathological severity score ≥3 corresponding to HSIL or invasive cervical cancer, whereas 83 had a score below 3 and were included in the negative outcome group. RLU/CO alone demonstrated moderate discrimination for identifying HSIL or invasive cervical cancer, with an AUC of 0.659 (95% bootstrap CI: 0.538–0.781). The exploratory model combining RLU/CO with HPV16/18 status yielded an AUC of 0.608 (95% bootstrap CI: 0.511–0.865), which was not significantly different from RLU/CO alone on paired DeLong testing (*P* = 0.618). The model combining RLU/CO with other high-risk HPV status had an AUC of 0.461 (95% bootstrap CI: 0.387–0.767) and did not differ significantly from RLU/CO alone (*P* = 0.074). The HPV16/18-integrated model had a higher AUC than the other high-risk HPV model (*P* = 0.049); however, neither genotype-integrated model demonstrated improved discrimination compared with RLU/CO alone. These findings are illustrated in Figure 4 and summarised in Table 4.

**Figure 4:**
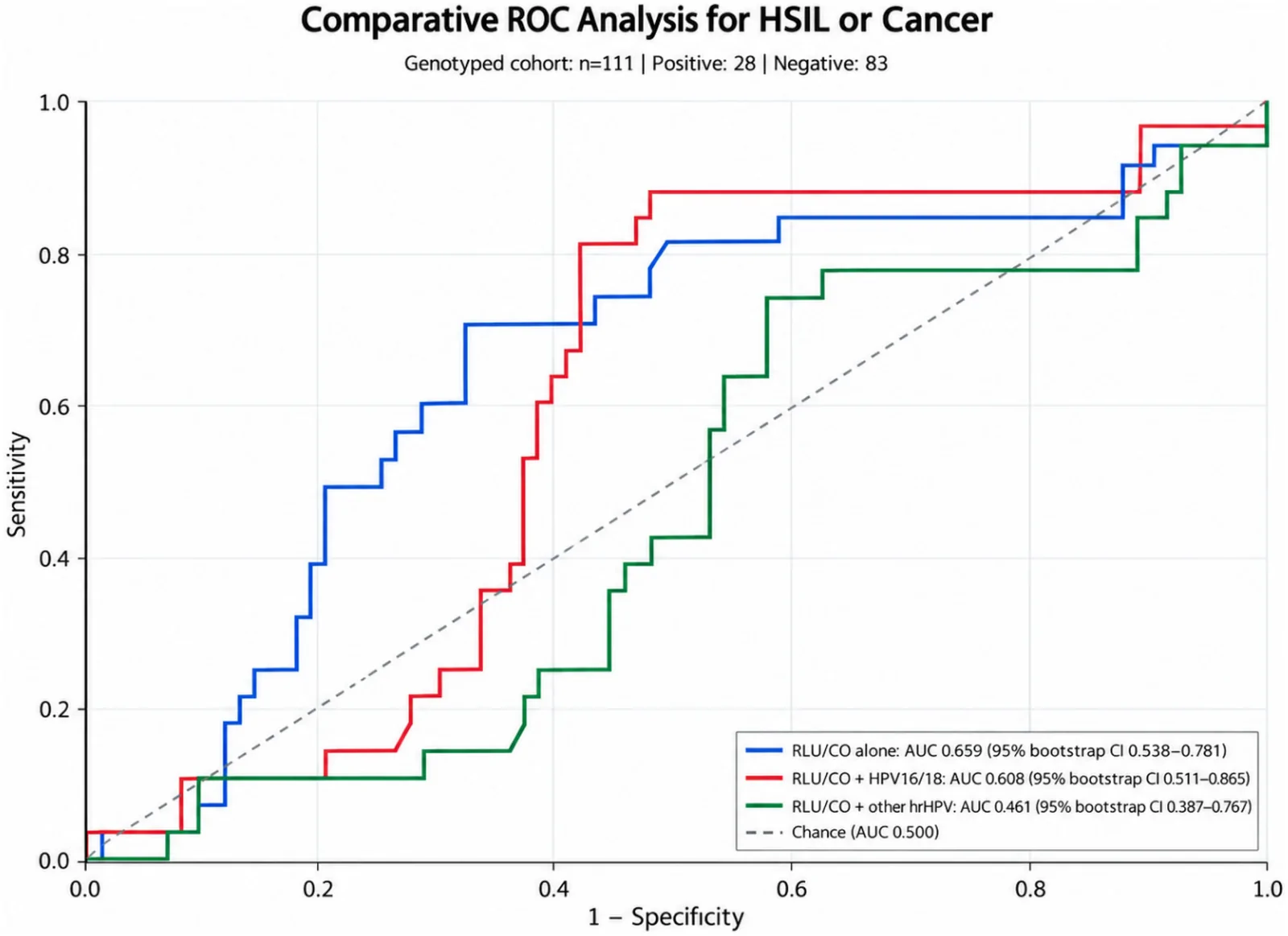
Comparative ROC analysis for identifying HSIL or invasive cervical cancer in the genotyped cohort. ROC curves compare RLU/CO alone (blue) with exploratory logistic-regression models combining RLU/CO with HPV16/18 status (red) or other high-risk HPV status (green) among 111 genotyped participants, including 28 with HSIL or invasive cervical cancer and 83 with a histopathological severity score below 3. The outcome was defined as a study-specific histopathological severity score of ≥3; LSIL (score 2) was included in the negative outcome group. RLU/CO alone achieved an AUC of 0.659 (95% bootstrap CI, 0.538–0.781); the RLU/CO plus HPV16/18 model had an AUC of 0.608 (95% bootstrap CI, 0.511–0.865); and the RLU/CO plus other high-risk HPV model had an AUC of 0.461 (95% bootstrap CI, 0.387–0.767). The diagonal dashed line represents chance-level discrimination. Because the models were estimated in a small subgroup without internal or external validation, results are exploratory and do not establish clinical utility.

**Table 4:** Comparative ROC performance for HSIL or invasive cervical cancer.

Model	AUC	95% CI	Pairwise comparison	*P* value
RLU/CO alone	0.659	0.538–0.781	Reference	—
RLU/CO + HPV16/18	0.608	0.511–0.865	Versus RLU/CO alone	0.618
RLU/CO + other high-risk HPV	0.461	0.387–0.767	Versus RLU/CO alone	0.074
RLU/CO + HPV16/18	0.608	0.511–0.865	Versus other high-risk HPV model	0.049

Analysis included 111 genotyped participants: 28 with HSIL or invasive cervical cancer (study-specific histopathological severity score ≥3) and 83 with a score <3; LSIL was included in the negative outcome group. Confidence intervals were obtained by bootstrap resampling. Paired DeLong tests were used to compare AUCs because all models were evaluated in the same participants. HPV16/18 and other high-risk HPV indicators were not mutually exclusive.

Because HPV16/18 and other high-risk HPV indicators were not mutually exclusive, participants with mixed infections could contribute to both genotype-integrated models. The comparisons therefore reflect the performance of different predictive models in the same genotyped cohort and should not be interpreted as comparisons between mutually exclusive infection groups. Given the small sample size, limited number of HSIL/cancer events, and absence of internal or external validation, these genotype-integrated findings should be considered exploratory and require confirmation in larger independent cohorts.

## 4 Discussion

The present study examined the relationship between viral load values and cervical lesion severity in a cohort of HPV-positive Indian women and explored whether genotype information may add value to triage assessment. In our cohort, RLU/CO values increased across the histopathological spectrum, with higher values observed in women with HSIL and invasive cancer than in those with normal histology or chronic cervicitis, replicating the results from previous studies (8, 9). This supports the view that HC2 viral load can serve as a practical semi-quantitative biomarker in HPV-positive women undergoing cervical evaluation. At the same time, the overlap in values across categories suggests that RLU/CO should be interpreted as a probabilistic marker rather than as a stand-alone diagnostic measure (10).

The boxplot analysis showed a clear shift toward higher log10(RLU/CO) values with severe histopathological grade, particularly in HSIL and cancer categories. The normal and chronic cervicitis groups were concentrated at lower values, while the higher-grade categories showed broader spread and higher central tendency. This pattern is clinically relevant because it suggests that viral load may help separate women with lower-grade findings from those with more concerning lesions, although overlap between groups remains substantial. The variation in viral load among high-grade lesions suggests that not all advanced cases behave similarly, making it difficult for a single cutoff value to accurately classify all women.

The cancer category warrants careful interpretation. Although the cancer group showed elevated RLU/CO values overall, the median (225.41) was lower than HSIL (242.04). This reflects the biological heterogeneity of invasive disease and the episomal-to-integrated HPV DNA transition during carcinogenesis, which reduces viral particle production. Similar findings have been reported where viral load increases from normal to HSIL but becomes inconsistent in cancer, with some studies showing lower loads in invasive disease (2, 6, 11). Thus, RLU/CO is useful for risk stratification but cannot strictly separate every high-grade lesion from cancer, supporting its use as part of a broader triage approach rather than a sole marker of disease stage.

In the full cohort, ROC analysis supported useful discriminatory performance for identifying clinically significant lesions, defined as a study-specific histopathological severity score of ≥3. The RLU/CO cutoff of 12.08 was selected and evaluated in the same cohort; therefore, its observed sensitivity, specificity, and overall diagnostic performance may be optimistic. The cutoff should be regarded as cohort-specific and exploratory, as its performance may vary with disease prevalence, case mix, specimen collection, assay conditions, and population characteristics. Independent external validation, ideally in prospective screening populations, is required before this threshold can be considered for clinical triage. Similarly, the genotype-integrated HPV16/18 model showed only modest apparent discrimination (AUC 0.608) in a small subgroup and did not improve performance over RLU/CO alone. This result does not establish clinical utility or justify a combined viral-load/genotype triage algorithm.

The study population was recruited from women undergoing diagnostic cervical evaluation after a positive HPV result rather than from a population-based screening programme. This referral setting, together with the use of colposcopy-guided biopsy in all included participants, likely enriched the cohort for clinically apparent disease and contributed to the relatively high proportion of invasive cervical cancer. Therefore, the observed prevalence of HSIL/cancer, RLU/CO distributions, and diagnostic-performance estimates may not be directly generalisable to routine primary-screening populations. The proposed RLU/CO cutoff should consequently be regarded as cohort-specific and exploratory, requiring validation in a prospective screening cohort before it can be considered for triage use.

Although several studies have reported higher HPV viral-load values in women with high-grade cervical lesions, the association has not been consistent across all studies. Some investigations have observed substantial overlap in viral-load measurements between low-grade and high-grade lesions, with limited ability of viral load alone to distinguish clinically significant disease. Other studies have reported variable or reduced viral-load levels in invasive cancer compared with HSIL, suggesting that viral load may be influenced by HPV genotype, episomal-to-integrated viral DNA transition, lesion biology, specimen cellularity, and assay-related factors (6, 12). Consequently, reported diagnostic performance and optimal RLU/CO thresholds have varied considerably across populations and testing platforms. These findings support interpreting viral load as a risk-stratification marker rather than a definitive diagnostic test.

The genotype findings from the 111-participant subgroup provide descriptive and exploratory information about HPV diversity across histopathological categories. HPV16 was the most frequently detected genotype, while other commonly observed types varied between categories. Mixed infections were frequent in the majority of the cohort, indicating that participants could harbour several HPV types simultaneously and that genotype patterns were not restricted to a single dominant type (13). These findings support the potential relevance of broader genotype characterisation; however, the small subgroup size and limited numbers within individual histopathological categories mean that the observed genotype distributions should be considered hypothesis-generating rather than definitive.

The comparative ROC analysis in the genotyped cohort did not demonstrate incremental diagnostic benefit from genotype integration. RLU/CO alone showed moderate discrimination for HSIL or invasive cervical cancer, with an AUC of 0.659. The model combining RLU/CO with HPV16/18 status had an AUC of 0.608 and did not significantly differ from RLU/CO alone, while the model combining RLU/CO with other high-risk HPV status had lower apparent discrimination, with an AUC of 0.461. Although the HPV16/18-integrated model performed better than the other-high-risk-HPV model, neither combined model improved discrimination over RLU/CO alone. Therefore, these findings do not support a clear added value of the evaluated genotype indicators for viral-load-based triage in this small subgroup. Given the limited sample size, number of outcome events, and absence of internal or external validation, the genotype-integrated analyses should be considered exploratory and should not be used to support a clinical triage algorithm.

## 5 Strengths

This study included a histopathologically characterised cohort of HPV-positive Indian women spanning normal histology, benign disease, LSIL, HSIL, and cancer. It evaluated RLU/CO both across lesion severity categories and through ROC analysis using a clinically relevant endpoint of HSIL or invasive cancer. The study also included exploratory genotype analysis, mutually exclusive descriptive genotype classification, and paired comparisons of genotype-integrated ROC models. Reporting confidence intervals and acknowledging the need for external validation strengthen the interpretation of the findings.

## 6 Limitations

Several factors limit interpretation of the genotype-integrated models. The analysis included only 111 genotyped participants, of whom 28 had HSIL or invasive cancer, and the genotype indicators were not mutually exclusive; therefore, participants with mixed infections could contribute to more than one genotype-based model. In addition, confidence intervals were relatively wide, reflecting the limited sample size, and the models were evaluated in the same cohort in which their performance was estimated. The findings should consequently be regarded as exploratory and supportive of further investigation rather than as conclusive evidence of clinical utility. Larger prospective studies with comprehensive genotype characterisation, adequate representation of high-grade lesions and cancer, prespecified modelling strategies, and independent external validation are required before viral-load and genotype-based triage can be recommended for clinical implementation.

## 7 Conclusion

In this cohort of HPV-positive Indian women, RLU/CO was associated with cervical lesion severity and demonstrated limited-to-moderate discrimination for identifying HSIL or invasive cervical cancer. Genotype-integrated analysis suggested that adding HPV16/18 status did not improve discrimination for HSIL or invasive cervical cancer compared with RLU/CO alone; however, the overall performance remained modest, and the model incorporating other high-risk HPV status showed limited discrimination. Given the small genotyped subgroup, these findings are exploratory and should not be interpreted as evidence for immediate clinical implementation. Larger prospective studies with comprehensive genotype characterisation and independent validation are needed to establish the reproducibility and clinical utility of viral-load and genotype-based triage before any clinical algorithm is proposed.

## Data Availability

The data analyzed in this study is subject to the following licenses/restrictions: Data is not available publicly due to institutional policy. Requests to access these datasets should be directed to nilanchalisingh@gmail.com.
